# The Effect of Nutritional Ketosis on Aquaporin Expression in Apolipoprotein E-Deficient Mice: Potential Implications for Energy Homeostasis

**DOI:** 10.3390/biomedicines10051159

**Published:** 2022-05-18

**Authors:** Inês V. da Silva, Sean Gullette, Cristina Florindo, Neil K. Huang, Thomas Neuberger, A. Catharine Ross, Graça Soveral, Rita Castro

**Affiliations:** 1 Research Institute for Medicines (iMed.ULisboa), Faculty of Pharmacy, Universidade de Lisboa, 1649-003 Lisbon, Portugal; imsilva1@campus.ul.pt; 2 Department of Pharmaceutical Sciences and Medicines, Faculty of Pharmacy, Universidade de Lisboa, 1649-003 Lisbon, Portugal; cristinaflorindo@ff.ulisboa.pt; 3 Huck Institutes of the Life Sciences, The Pennsylvania State University, State College, PA 16802, USA; sqg5746@psu.edu (S.G.); tun3@psu.edu (T.N.); 4 Department of Nutritional Sciences, The Pennsylvania State University, State College, PA 16802, USA; neil.huang@tufts.edu (N.K.H.); acr6@psu.edu (A.C.R.); 5 Cardiovascular Nutrition Laboratory, Jean Mayer USDA Human Nutrition Research Center on Aging, Tufts University, Boston, MA 02111, USA; 6 Department of Biomedical Engineering, The Pennsylvania State University, University Park, State College, PA 16802, USA

**Keywords:** aquaporins, very low-carbohydrate diet, ketogenic diet, obesity

## Abstract

Ketogenic diets (KDs) are very low-carbohydrate, very high-fat diets which promote nutritional ketosis and impact energetic metabolism. Aquaporins (AQPs) are transmembrane channels that facilitate water and glycerol transport across cell membranes and are critical players in energy homeostasis. Altered AQP expression or function impacts fat accumulation and related comorbidities, such as the metabolic syndrome. Here, we sought to determine whether nutritional ketosis impacts AQPs expression in the context of an atherogenic model. To do this, we fed *ApoE^−/−^* (apolipoprotein E-deficient) mice, a model of human atherosclerosis, a KD (Kcal%: 1/81/18, carbohydrate/fat/protein) or a control diet (Kcal%: 70/11/18, carbohydrate/fat/protein) for 12 weeks. Plasma was collected for biochemical analysis. Upon euthanasia, livers, white adipose tissue (WAT), and brown adipose tissue (BAT) were used for gene expression studies. Mice fed the KD and control diets exhibited similar body weights, despite the profoundly different fat contents in the two diets. Moreover, KD-fed mice developed nutritional ketosis and showed increased expression of thermogenic genes in BAT. Additionally, these mice presented an increase in Aqp9 transcripts in BAT, but not in WAT, which suggests the participation of Aqp9 in the influx of excess plasma glycerol to fuel thermogenesis, while the up-regulation of *Aqp7* in the liver suggests the involvement of this aquaporin in glycerol influx into hepatocytes. The relationship between nutritional ketosis, energy homeostasis, and the AQP network demands further investigation.

## 1. Introduction

Despite significant advances in atherosclerotic cardiovascular disease (CVD) treatment, CVD remains the leading cause of mortality among adults [1]. The mechanistic underpinnings of dietary pattern-related CVD risk are still not well understood. An apolipoprotein E-deficient (*ApoE*^−/−)^ mouse model is a pre-clinical model that is widely used to study the pathophysiology of vascular plaque formation and atherosclerosis [2,3]. The global obesity epidemic is one of the main risk factors for atherosclerosis and CVD [4]. Recent scientific evidence highlights the ketogenic diet (KD)—a very low-carbohydrate, high-fat diet—as a promising strategy to treat obesity [5]. Interestingly, this diet was initially used to treat refractory epilepsy in children [6] and is now being tested as a dietary approach to treat other disorders, such as a common renal pathology (autosomal dominant polycystic kidney disease) [7,8,9]. The fundamental principle of the KD is a severe restriction of dietary carbohydrate consumption with a concurrent increase in dietary fat consumption to compensate for the energy deficit, resulting in a metabolic state of nutritional ketosis. The impact of a KD on cardiometabolic and vascular health is still a subject under intense debate [10,11]. In fact, in being used to achieve ketosis, KDs are typically inconsistent with nutritional recommendations for CVD prevention; cardio-protective foods are severely restricted (e.g., fruits, legumes) and foods associated with increased CVD risk are promoted (e.g., meats rich in saturated fat) [12,13]. 

The unique macronutrient profile of the KD results in the promotion of lipid oxidation to produce ketones as an energy source [12]. In fact, this metabolic adaptation is based on coordinated responses of the liver and an altered energetic metabolism at the cellular level. When there is an adequate supply of carbohydrates, cells primarily rely on glucose metabolism, whereas under carbohydrate-depletion conditions, cells use ketone bodies as their primary energy source [14,15]. Ketone bodies are produced in the liver via mitochondrial β-oxidation and are then released into the blood for uptake and utilization by peripheral tissues. Moreover, a critical metabolic change in response to a KD involves the mobilization of lipids stored in adipose tissue, with triacylglycerols (TAG) being hydrolyzed to yield glycerol and fatty acids to be taken up by the liver to feed hepatic ketogenesis [16].

Several proteins have been identified as key players in energy balance and vascular homeostasis. Aquaporins (AQPs) are transmembrane proteins that function as channels, allowing the permeation of water, glycerol, and other small non-charged molecules across biological membranes driven by osmotic or solute gradients [17]. Thirteen AQP isoforms have been identified in humans (AQP0–AQP12) and they are widely distributed in tissue-specific manners [18]. AQPs have been categorized into three subgroups, according to their transport selectivity and primary structure: classical aquaporins (AQP0, AQP1, AQP2, AQP4, AQP5, AQP6, and AQP8), which are primarily selective to water; aquaglyceroporins (AQP3, AQP7, AQP9, and AQP10), which also facilitate the permeation of other small solutes (glycerol, urea); and S-aquaporins (AQP11 and AQP12), comprising intracellular isoforms that have impacts on organelle homeostasis [19,20]. Recently, a few isoforms have also been reported to transport hydrogen peroxide and have been termed peroxiporins (AQP1, AQP3, AQP5, AQP8, AQP9, and AQP11) [21,22,23], opening new perspectives in understanding the potential roles of AQPs in physiological redox balance as well as oxidative stress [24,25].

The involvement of aquaglyceroporins in adipose tissue homeostasis has possible implications for metabolic disorders [26]. In fasting conditions, glycerol from TAG lipolysis in adipose tissue is released via AQP7 into the bloodstream and is taken up via AQP9 by the liver as a substrate for gluconeogenesis [27]. The adipose AQP7–hepatic AQP9 axis has been extensively characterized, and the synchronization of these AQPs ensures glycerol metabolism for gluconeogenesis [28]. In addition, the involvement of AQPs in obesity-induced inflammation [29], as well as their ability to trigger inflammatory processes involved in metabolic disorders [30,31], suggests their importance in the maintenance of vascular homeostasis.

In this study, we have investigated how nutritional ketosis induced by a KD diet in the context of an atherogenic model impacts the transcript levels of AQPs involved in energy homeostasis. We fed *ApoE*^−/−^ mice a KD or control diet for 12 weeks and assessed metabolic disturbances. After confirming the presence of nutritional ketosis in the KD-fed mice versus the control group, we performed gene expression studies for key metabolic tissues.

## 2. Materials and Methods

### 2.1. Animals

Male *ApoE*^−/−^ mice were purchased from Jackson Laboratory (Bar Harbor, ME, USA) and housed in a temperature- and humidity-controlled room. Only male mice were included to control for the known effect of sex on atherosclerosis in this strain [32]. Wild-type mice, known to be resistant to atherosclerosis, were not included because we wanted to specifically address the consequences of a ketogenic diet in the context of an atherosclerosis-prone model [2]. At the age of 7 weeks, mice were weighed and divided into two groups (7–8 animals/group). All procedures were performed in compliance with the Institutional Animal Care and Use Committee of Pennsylvania State University (PRAMS#201747911 to R.C.), which specifically approved this study. 

### 2.2. Diets and Feeding

Mice were fed, for 12 weeks, one of the following diets based on AIN 93G [33] (Research Diets, New Brunswick, NJ, USA) with modifications (Kcal%; fat/carbohydrate/protein): a very-low-carbohydrate KD diet (81/1/18) with 0.15% cholesterol or a control diet (12/70/18). Details about diet composition are shown in Table 1. Diets were replaced once a week, at which time the animals and the remaining food were weighed to determine food consumption and body weight progression. 

### 2.3. Blood Sampling and Measuring of Biochemical/Metabolic Markers

Every four weeks, approximately 200 µL of blood was collected from the retroorbital cavity into heparinized tubes and immediately placed on ice. Plasma was isolated by centrifugation at 4 °C and immediately stored at −80 °C prior to biochemical analyses.

#### 2.3.1. β-Hydroxybutyrate, Triacylglycerols, Total Cholesterol, and Glucose

Plasma collected at 8 weeks was tested for ß-hydroxybutyrate (BHB), TAG, and total cholesterol contents using colorimetric assays kits (Randox, Ann Arbor, MI, USA) following the manufacturer’s instructions. Blood glucose was measured using a glucometer (Contour, Bayer, Tarrytown, NY, USA) following the manufacturer’s instructions.

#### 2.3.2. Glutathione Amino Acids Precursors

In plasma collected at 12 weeks, the circulating levels of glutamate and glycine were determined by gas chromatography–flame ionization detector (GC-FID) using the Phenomenex EZ:faastTM kit for physiological amino acid analysis (Phenomenex, Torrance, CA, USA) as previously described [34]. Moreover, plasma cysteine concentrations were quantified by high-performance liquid chromatography (HPLC) analysis with fluorometric detection, as previously described [35].

### 2.4. Tissue Collection 

After 12 weeks, mice were euthanized by carbon dioxide inhalation. Aortas were obtained and subjected to Oil Red O staining, as previously described in detail [36]. Livers were removed and immediately snap-frozen in liquid nitrogen and stored at −80 °C. Inter-scapular brown adipose tissue (BAT) and epididymal white adipose tissue (WAT) were dissected following the protocol previously described by Bagchi and MacDougald [37], immediately snap-frozen in liquid nitrogen, and stored at −80 °C.

### 2.5. Quantification of Inflammatory Cytokines and Aortic Atheroma 

The effects of the experimental diets at 4 and 12 weeks on the plasma concentrations of tumor necrosis factor-alpha (TNF-α) and interleukin 6 (IL-6) were evaluated using ELISA assays (Meso Scale Diagnostics, Rockville, MD, USA), following the manufacturer’s instructions. 

An advanced imaging technique (14 T magnetic resonance imaging (MRI)) was used to quantify the volume of vascular lesions in the mouse aortas, as previously described in detail [36,38,39]. 

### 2.6. RNA Extraction 

Total RNAs were extracted from WAT, BAT, and liver tissue using the Qiagen RNeasy lipid tissue mini kit and Qiagen RNeasy mini kit, respectively (Qiagen, Germantown, MD, USA), followed by a DNAase treatment. All procedures were conducted following the manufacturer’s protocols. RNA concentrations were determined using the NanoDrop 2000c (Thermo Fisher Scientific, Waltham, MA, USA). Only samples with 260/280 nm ratios between 1.8 and 2.2 were used for cDNA synthesis. Additionally, agarose bleach gels were used to confirm RNA integrity, as previously described [40]. 

### 2.7. Quantitative PCR Analysis

Reverse transcription of 1µg RNA was performed using M-MLV reverse transcriptase and oligo dT primers (Promega, Madison, WI, USA). Amplification by quantitative PCR was executed using a CFX96 Real-Time System C1000 (BioRad, Hercules, CA, USA) after cDNA was mixed with TaqMan Universal PCR Master Mix and the following specific TaqMan gene expression assays (Applied Biosystems, Foster City, CA, USA) were performed, as described in the manufacturer’s protocol and previous publications of our group [41]. The following probes and primers were used in this study: *Aqp1* (#Mm00431834_m1), *Aqp3* (#Mm01208559_m1), *Aqp5* (#Mm00437578_m1), *Aqp7* (#Mm00431839_m10), *Aqp9* (#Mm00508094_m1), *Ucp1* (#Mm01244861_m1), and *Eef2* (#Mm00833287_g1). Relative gene expression was calculated using a variation of the Livak method with a houskeeping gene *Eef2* normalization step [42,43].

### 2.8. Statistical Analyses

Analyses were performed in GraphPad Prism 7 (GraphPad Software, La Jolla, CA, USA), with statistical significance set to *p* < 0.05. For comparison of two groups, an unpaired Student’s *t*-test was used. 

## 3. Results

### 3.1. KD-Fed Mice Weight Gain Was Similar to Controls 

**Figure 1:**
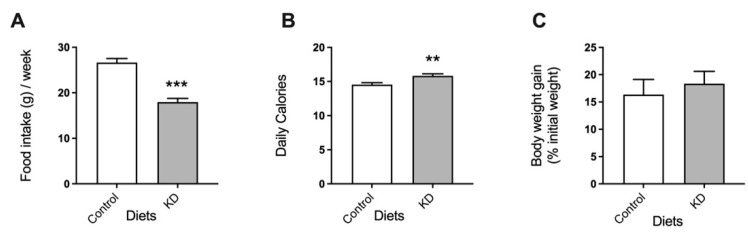
The effect of the experimental diets on (**A**) food consumption, (**B**) calories consumed, and (**C**) body weight. Data shown are the mean ± SEM, *n* = 7–8/group. **, *p* < 0.01; ***, *p* < 0.001, KD versus control.

KD-fed mice consumed significantly less food than mice fed the control diet (Figure 1A). Nevertheless, due to the higher energy density of this diet (6.2 kcal/g KD versus 3.9 kcal/g control diet), KD-fed mice consumed more calories than the controls (Figure 1B). Notably, despite the higher number of calories consumed by the KD group than the controls, both groups of mice presented similar body weights (Figure 1C).

### 3.2. KD Promoted Nutritional Ketosis and a Distinct Metabolic Profile 

**Figure 2:**
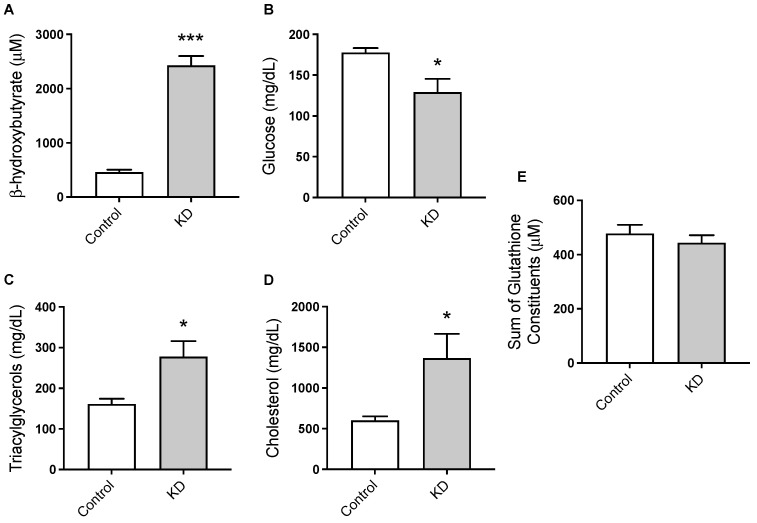
The effect of the experimental diets on the circulating levels of (**A**) ß-hydroxybutyrate, (**B**) glucose, (**C**) triacylglycerols, (**D**) total cholesterol, and (**E**) the sum of glutamate, glycine, and cysteine. Data shown are the mean ± SEM, *n* = 7–8/group. *, *p* < 0.05; ***, *p* < 0.001, KD versus control.

The plasma concentration of the major ketone body, ß-hydroxybutyrate (BHB), was significantly elevated in KD mice, confirming the presence of nutritional ketosis under this dietary condition (Figure 2A). Specifically, BHB concentrations (μM, mean ± SEM) were 2427 ± 347 for KD and 460 ± 85 for control mice. On the other hand, plasma TAG (Figure 2C) and total cholesterol levels (Figure 2D) were significantly increased by the KD (Figure 2D). Blood glucose levels showed opposite results, with KD-fed mice presenting significantly lower blood glucose concentrations than the controls (Figure 2B). We also assessed the effect of the KD on the sum of plasma glutamate, glycine, and cysteine, which determine glutathione availability [44]. No significant differences were detected between the two groups of animals (Figure 2E). Glutathione is a tripeptide that constitutes the most abundant intra-cellular antioxidant system and it is a major determinant of redox balance. Thus, this observation suggests that glutathione availability was preserved in KD mice.

### 3.3. KD Augmented Plasma TNF-α and IL-6 and Vascular Lesions

**Figure 3:**
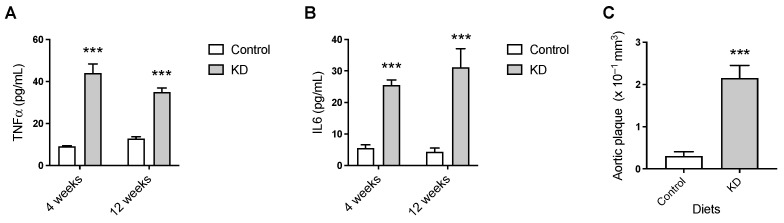
The effect of 12 weeks of KD on systemic inflammation and atherosclerotic plaque burden. Plasma concentrations of (**A**) interleukin 6 (IL-6) and (**B**) tumor necrosis factor α (TNF-α). (**C**) Ex vivo 14T-MRI volumetric assessment of total atheroma. Data shown are the mean ± SEM, *n* = 4–8/group. ***, *p* < 0.001, KD versus control.

We next examined the effect of nutritional ketosis on the circulating levels of the pro-inflammatory cytokines TNF-α and IL-6 and on the volume of atheroma. The results suggested a sustained pro-inflammatory effect of the KD when compared to the control diet. Specifically, feeding the experimental diets for as little as 4 weeks resulted in significantly higher plasma concentrations of TNF-α and IL-6 in the KD mice than in the controls, which were maintained at 12 weeks (Figure 3A,B). Moreover, levels of aortic atherosclerotic plaque in KD-fed mice, as assessed by MRI, were significantly elevated compared to control levels (0.23 ± 0.03 versus 0.03 ± 0.01 mm^3^, mean ± SEM, *n* = 7–8) (Figure 3C). 

### 3.4. KD Up-Regulated the Expression of Thermogenic Genes

**Figure 4:**
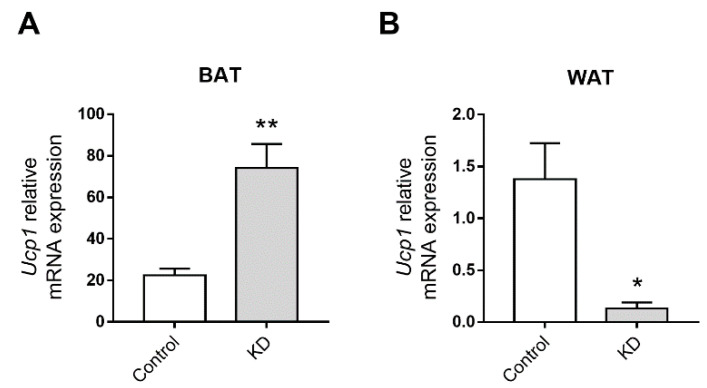
Effect of the ketogenic diet (KD) on the transcription levels of Uncoupling protein 1 (Ucp1) in (**A**) BAT and (**B**) WAT of ApoE^−/−^ mice fed the experimental diets for 12 weeks. Data shown are the mean ± SEM (*n* = 6–8/group). *, *p* < 0.05; **, *p* < 0.01, KD versus control.

The transcript level of Ucp1 in BAT of KD-fed mice was four times higher compared to control mice (Figure 4A), revealing a thermogenic effect of the KD. In contrast, the transcript level for Ucp1 in WAT was significantly decreased compared to the controls (around a 0.15-fold change; Figure 4B).

### 3.5. KD Altered AQPs Expression in Adipose Tissues and Liver

**Figure 5:**
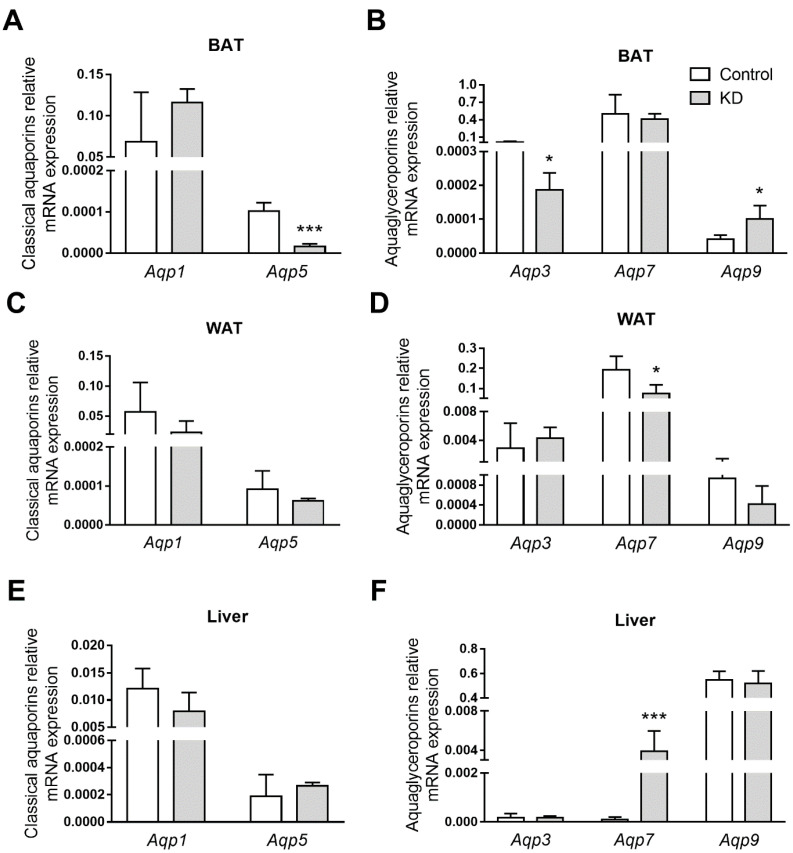
Aquaporin gene expression in (**A**,**B**) white adipose tissue (WAT), (**C**,**D**) brown adipose tissue (BAT), and (**E**,**F**) the livers of *ApoE*^−/−^ mice fed control or a ketogenic diet (KD). Relative gene expression of (**A**,**C**,**E**) classical aquaporins and (**B**,**D**,**F**) aquaglyceroporins. Data shown are the mean ± SEM (*n* = 6–8/group). *, *p* < 0.05; ***, *p* < 0.001, KD versus control.

The gene expression of aquaporins implicated in endothelial homeostasis (Aqp1 and Aqp5) [45] and energetic metabolism (Aqp3, Aqp7, and Aqp9) [26] were evaluated in BAT, WAT, and the liver, which are tissues mainly involved in energy homeostasis. All the AQP isoforms were detected in all the investigated tissues, which is in accordance with previous reports [38]. However, each AQP showed a tissue-specific profile (Figure 5). For example, in BAT, high Aqp7 and Aqp1 transcripts levels were detected, while Aqp3, Aqp5, and Aqp9 were present in lower amounts (Figure 5A,B). As with BAT, WAT contained abundant levels of Aqp7 and Aqp1 transcripts, followed by lower expression of Aqp3, Aqp9, and Aqp5 (Figure 5C,D). In the WAT of mice fed the KD, Aqp7 mRNA levels were low, but the expression of the other isoforms was not altered by the diets. The BAT of these animals, however, presented *Aqp9* up-regulation. Further, this same isoform was the most representative aquaglyceroporin in the liver, as previously reported [46], but expression of Aqp1, Aqp3, Aqp7, and Aqp5 was also detected (Figure 5E,F). In KD-fed mice, an intense 20-fold hepatic induction of Aqp7 transcript was also observed.

## 4. Discussion

In recent years, KDs have been considered a promising strategy to treat obesity—a major CVD risk factor. KDs contain 70–80% of kcal from fat but very little carbohydrate, which stimulates endogenous ketogenesis, thus yielding high levels of BHB. Previous studies with mice have reported an anti-obesogenic effect for the KD, despite its extremely high fat content [47,48,49,50]. Accordingly, in this study, notwithstanding a caloric intake that was markedly higher in KD-fed mice than in controls and 12 weeks of ad libitum feeding, no significant differences were observed in body weight between KD- and control-fed *ApoE*^−/−^ mice. As we wanted to focus on atherogenesis, we did not include wild-type mice, which are not atherosclerosis-prone. However, we acknowledge that additional studies in wild-type mice could be of interest regarding other health outcomes. 

The absence of dietary carbohydrates in KD stimulates the hepatic production of ketone bodies that are further transported to extrahepatic tissues for terminal oxidation as the primary energy source [51]. In this study, the plasma concentration of the major ketone body, BHB, was significantly elevated in KD-fed mice and within the range reported by others [49,51]. This finding confirms the presence of nutritional ketosis in KD-fed mice and indicates the reliability of this dietary mouse model in understanding the effect of nutritional ketosis on aquaporin expression. On the other hand, and as previously reported, KD promoted an accumulation of circulating TAG and cholesterol levels together with significantly lowered blood glucose levels compared to controls [52,53,54]. 

The ratio of the concentration of the universal methyl donor S-adenosylmethionine (AdoMet) to S-adenosylhomocysteine (AdoHcy) (AdoMet:AdoHcy) is an index of cellular transmethylation reactions [55]. In previous cell studies, dysregulation of the transmethylation capacity impacted *AQP1* expression and promoted an atherogenic phenotype [45]. Moreover, an increased dietary fat content in the presence of carbohydrates was associated with a diminished systemic methylation index in wild-type mice [56] but not in *ApoE*^−/−^ mice [38]. The effect of the diets used in this study on AdoMet:AdoHcy ratio has recently been published [39]. A significantly decreased plasma AdoMet:AdoHcy ratio was detected in KD-fed *ApoE*^−/−^ mice versus controls, revealing systemic hypomethylation under nutritional ketosis. This observation led us to focus in the present study on the glutathione system. In fact, a link between hypomethylation and oxidative stress due to the dysregulation of the antioxidant glutathione system has been previously reported [57]. Moreover, disturbances in redox balance contribute to susceptibility and pathology in human diseases, including atherosclerosis. Interestingly, cumulative evidence points to the role of AQPs as facilitators of ROS membrane permeation [58]. These intriguing observations led us to assess the effect of the KD on the sum of glutamate, glycine, and cysteine concentrations. Nevertheless, no significant differences were detected between the two groups of animals, suggesting that glutathione availability was preserved with the KD.

Inflammation plays an important role in the progression of atherosclerosis [59]. Furthermore, compelling evidence strongly suggests that AQPs are key regulators of inflammation [31]. The pro-inflammatory cytokines TNF-α and IL-6 play crucial roles in inflammation and exacerbate atherosclerosis in murine species [60,61,62]. The results suggested a sustained pro-inflammatory effect of the KD when compared to the control diet. As previously mentioned, the KD contains approximately 80% of Kcal from fat. Thus, although other cytokines could have been quantified in plasma to unequivocally address and compare the levels of systemic inflammation between the KD and control groups, our data are consistent with the well-established positive correlation of dietary fat and systemic inflammation [63]. KD-fed mice developed more extensive atherosclerosis than control mice, which may have been contributed to, in part, by elevated systemic inflammation in the KD mice. Previous studies in which high-fat diets with very low carbohydrate contents were fed to murine models of atherosclerosis also reported atherogenic effects of the diets [53,64]. In the present study, our goal was to test whether a KD, in the context of an atherogenic model, impacted AQPs expression. Thus, we used a diet that was very low in carbohydrates, very high in fat, and contained 0.15% of cholesterol, this being typical of atherogenic diets described in the literature. We acknowledge that cholesterol, as a well-established dietary atherogenic component in *ApoE*^−/−^ mice, may have contributed to the observed KD-induced exacerbated systemic inflammation and vascular toxicity [65,66]. Thus, future studies conducted without dietary cholesterol are needed to unequivocally address the vascular effect of nutritional ketosis in this mouse model. In fact, increasing evidence suggests a positive impact of nutritional ketosis on vascular physiology and homeostasis in humans [67,68,69]. 

The finding of similar body weight in all mice, despite the higher caloric intake in the KD-fed group, was intriguing. BAT plays a vital role in energy homeostasis and heat production [70]. The thermogenic function of BAT is dependent on Ucp1, a protein expressed in the inner mitochondrial membrane of brown adipocytes [71]. Ucp1 disrupts the electrochemical gradient across the inner mitochondrial membrane, causing energy derived from the metabolism of food to be released as heat [71]. The exceptional function of BAT to increase energy expenditure is shown by its anti-obesogenic role. In fact, mice deficient in Ucp1 are susceptible to weight gain [72], whereas an excess of Ucp1 protects against diet-induced obesity [73]. In the present study, *Ucp1* transcription in BAT of KD-fed mice was observed to be up-regulated as compared to the controls. Thus, it is tempting to speculate that this KD-induced up-regulation of *Ucp1* may have contributed to the similar body weight of KD-fed mice and controls despite the first group having a higher caloric intake. Additional studies assessing basal metabolism are warranted to explore this exciting possibility. 

Lastly, it was observed that the transcriptional levels of several AQPs were different among the tissues involved in energy homeostasis, namely, the liver, BAT, and WAT. Further, and interestingly, this tissue-specific profile was altered when mice were subjected to KD (Figure 5). In BAT, KD induced a two-fold increase in Aqp9 expression, suggesting that this isoform provides a route for the influx of excess plasma glycerol to be used as fuel in thermogenesis. Thermogenesis is a very intensive process in terms of energy; thus, BAT has a high metabolic demand. Recent studies have identified the need for a variety of metabolic substrates to initiate and maintain thermogenesis, such as intracellular triglycerides and glucose, in addition to the well-established free fatty acids [74]. The high expression of BAT glycerol kinase, the enzyme that converts glycerol to glycerol-3-phosphate for triacylglycerol synthesis in both rodents and humans [75], suggests an important role for glycerol in maintaining intracellular levels of TAG during thermogenesis and highlights the role of AQPs in the process. In addition, KD impaired Aqp3 and Aqp5 expression, both peroxiporins acting as hydrogen peroxide facilitators, suggesting that they might be involved in an unbalanced redox potential induced by KD. Interestingly, the same AQP response pattern was observed in our previous study with mice fed a high-fat diet with a higher carbohydrate content [38], suggesting that high-calorie diets affect BAT similarly independently of their macronutrient compositions. In the WAT of mice fed the KD, Aqp7 mRNA levels were low, suggesting that, due to increased circulating TAG, adipocyte lipid droplets were not used as fuel in KD-fed mice. AQP7 is the main gateway for glycerol efflux from white adipocytes following the breakdown of TAG. Finally, in KD-fed mice, an intense up-regulation of hepatic *Aqp7* was observed, which may indicate an additional glycerol uptake route as a compensatory strategy favoring gluconeogenesis. 

## 5. Conclusions

The current study contributes to a better characterization of an atherogenic-susceptible *ApoE*^−/−^ mouse model by reporting that the pattern of AQPs expression in these mice is disturbed in a tissue-specific manner by nutritional ketosis, which, in turn, was found to be associated with the up-regulation of thermogenic genes in BAT. Our data represent the first experimental approach ever reported on the modulation of AQPs expression in *ApoE*^−/−^ mice under this metabolic condition and warrant further investigation to establish and validate the relationship between nutritional ketosis, the AQP network, and energy homeostasis.

## Figures and Tables

**Table 1:** Macronutrients in the experimental diets.

Macronutrient (g/Kg of Diet)	Control Diet	Ketogenic Diet
Casein	180	280
Corn Starch	430	0
Maltodextrin 10	155	0
Sucrose	100	0
Cocoa Butter	0	155
Corn Oil	25	40
Primex (Non-Trans-Fat)	25	365

## Data Availability

Not applicable.
